# Adoption and impact of non-pharmaceutical interventions for COVID-19

**DOI:** 10.12688/wellcomeopenres.15808.1

**Published:** 2020-04-02

**Authors:** Natsuko Imai, Katy A.M. Gaythorpe, Sam Abbott, Sangeeta Bhatia, Sabine van Elsland, Kiesha Prem, Yang Liu, Neil M. Ferguson

**Affiliations:** 1 MRC Centre for Global Infectious Disease Analysis, Imperial College London, London, UK; 2 Centre for Mathematical Modelling of Infectious Diseases, London School of Hygiene & Tropical Medicine, London, UK

**Keywords:** non-pharmaceutical interventions, COVID-19

## Abstract

**Background**: Several non-pharmaceutical interventions (NPIs) have been implemented across the world to control the coronavirus disease (COVID-19) pandemic. Social distancing (SD) interventions applied so far have included school closures, remote working and quarantine. These measures have been shown to have large impacts on pandemic influenza transmission. However, there has been comparatively little examination of such measures for COVID-19.

**Methods**: We examined the existing literature, and collated data, on implementation of NPIs to examine their effects on the COVID-19 pandemic so far. Data on NPIs were collected from official government websites as well as from media sources.

**Results**: Measures such as travel restrictions have been implemented in multiple countries and appears to have slowed the geographic spread of COVID-19 and reduced initial case numbers. We find that, due to the relatively sparse information on the differences with and without interventions, it is difficult to quantitatively assess the efficacy of many interventions. Similarly, whilst the comparison to other pandemic diseases such as influenza can be helpful, there are key differences that could affect the efficacy of similar NPIs.

**Conclusions**: The timely implementation of control measures is key to their success and must strike a balance between early enough application to reduce the peak of the epidemic and ensuring that they can be feasibly maintained for an appropriate duration. Such measures can have large societal impacts and they need to be appropriately justified to the population. As the pandemic of COVID-19 progresses, quantifying the impact of interventions will be a vital consideration for the appropriate use of mitigation strategies.

## Introduction

As of the 21 March 2020, over 271,364 cases of coronavirus disease (COVID-19) have been confirmed globally across 174 countries and regions ^1^. Sustained human-to-human transmission has now been observed in multiple countries outside of mainland China including Italy, Japan, and South Korea with 47,021, 1,007, and 8,799 cases reported respectively ^1^. Conversely, some countries such as Bangladesh have more recently reported their first cases of COVID-19 resulting from importations of infected travellers from affected areas. In response, countries and regions have implemented a wide range of non-pharmaceutical interventions (NPIs). These NPIs have generally been scaled up over time in response to the magnitude of the outbreak in each country ^2^. NPIs can be broadly categorised into: i) personal protective measures such as hand hygiene; ii) environmental measures such as disinfection and ventilation; iii) social distancing measures such as school and workplace closures; and iv) travel related measures such as travel restrictions ^3^. As the first cases were exported from Wuhan City, China to countries and regions outside mainland China, early efforts focused on containment where infected individuals were rapidly identified and isolated ^4^. Contact tracing and active case finding efforts then identified any contacts potentially at risk of infection who were themselves isolated or monitored. Containment efforts thus focused on stopping transmission completely to prevent any community transmission ^5^. As case numbers increased and evidence of community transmission became apparent, countries and regions started to introduce a wider range of control measures including travel restrictions, improving public awareness through mass communication, widening surveillance efforts, distributing face masks, and social distancing (SD) measures ^6^.

SD measures can be effective control measures in outbreak settings ^7^. These can be broadly defined as: i) isolation, the separation of ill individuals from susceptible individuals; ii) quarantine, the separation of individuals who have been assumed to be exposed and; iii) community containment, an intervention applied to an entire community aimed at reducing contacts and movements including school and workplace closures and restrictions or cancellation of mass gatherings ^4^. Social distancing measures are intended to reduce mixing and rates of contact between individuals in the community, therefore reducing rates of potential transmission to the susceptible population ^8^.

It is important to note that control measures implemented during an epidemic are usually layered with other interventions and are often targeted. As countries and regions start to move towards mitigating the impact of the epidemic, measures are likely to be implemented to varying degrees. In this study, we focus on the use and implementation of social distancing measures in the current COVID-19 pandemic.

## Methods

### Interventions implemented

We extracted the date and type of SD interventions implemented in Wuhan (Hubei, China), South Korea, Japan, Hong Kong (Special Administrative Region of China), Singapore, and Italy. Apart from Wuhan, the other countries and regions were chosen as they were among the first or most affected places outside of mainland China (at the time of analysis) in the COVID-19 pandemic ^9^.

Relevant government websites such as ministry of health, ministry of education, and ministry of trade were identified through web searches. Information on interventions and the date they were implemented were extracted. We then supplemented these data using web searches with information from media reports on NPIs implemented in each country (see supplemental Table 1, extended data ^10^). We categorised the SD measures into 7 broad categories as summarised in Table 1. Information and dates of other NPIs, aside from SD measures, implemented early on in the epidemic such as travel advisories were also extracted (Supplemental Table 1, extended data ^10^).

**Table 1.** Summary of social distancing measures considered and/or implemented in response to the COVID-19 epidemic.

Measure	Description
Contact tracing	Identifying individuals who might have been in contact with a confirmed case.
Isolation	Separation of ill persons with contagious diseases from susceptible persons.
Quarantine	Restriction of persons who are presumed to have been exposed to a contagious disease but are not ill, either because they did not become infected or because they are still in the incubation period or because they did not become infected
School closures	Closure of schools nationally or across a region. This is distinct from reactive closures of schools in response to identified cases.
Workplace closure and measures	Closure of workplaces and advisories to work remotely.
Crowding	Advisories to avoid crowded places such as concerts. This includes mandatory cancellations of mass gatherings such as conferences, weddings, and funerals.
University closure	Regional or nationwide closure of universities.

### Analysis

Data on NPIs and SD measures were categorized manually and analysed using R version 3.6.2 ^11^. Replication code is available as extended data ^12^. Output data is available as underlying data ^12^.

## Results

**Figure 1.**
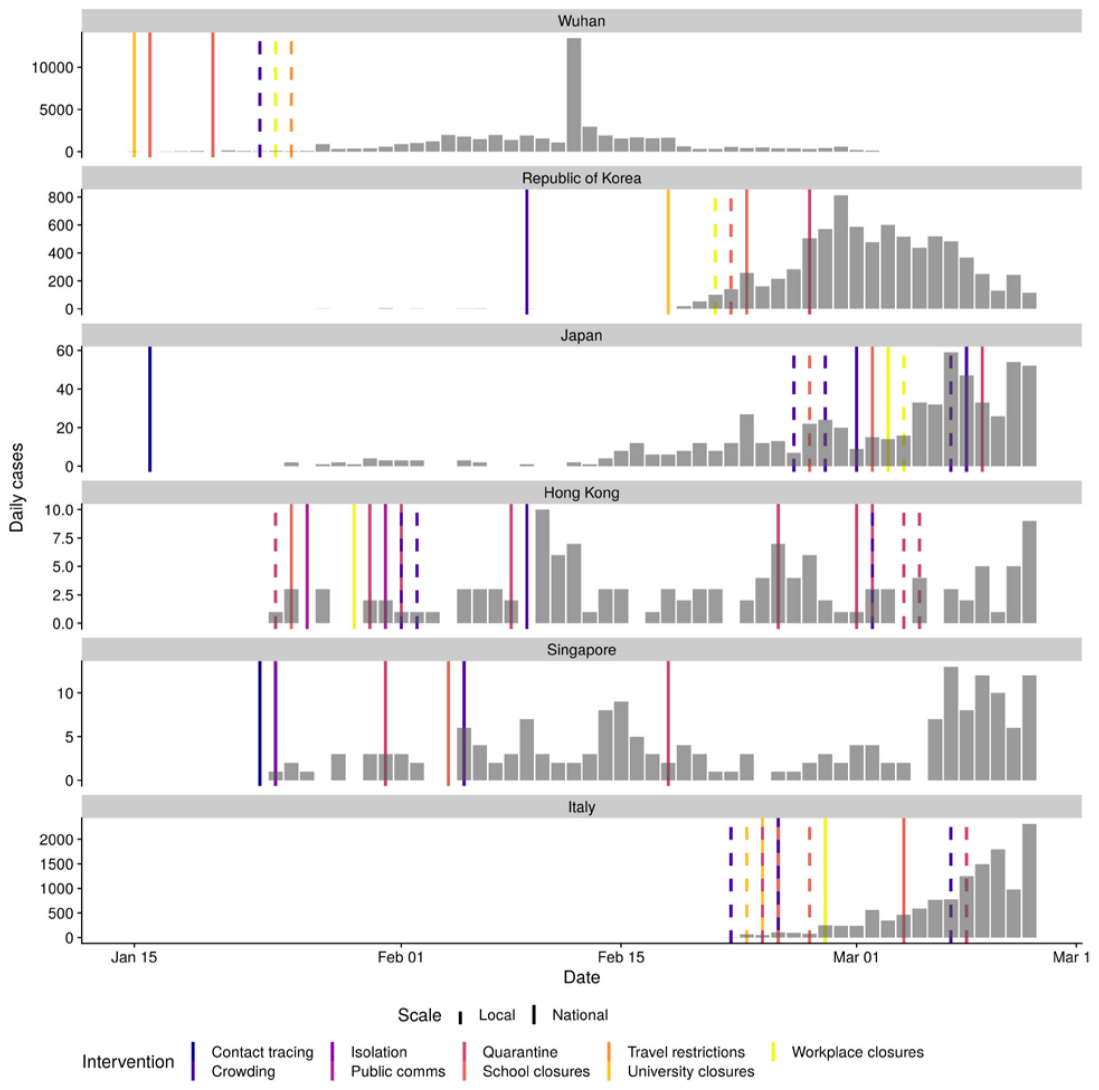
Number of cases by date of report for the five regions or countries with the highest number of cases outside of mainland China and Wuhan City as reported by WHO (taken from the WHO situational reports and Hubei Health Commission press releases). Note cases in Japan do not include the international conveyance. Each line represents the date of implementation of a social distancing measure. Note that some countries or regions had travel advice in place in response to the growing epidemic in China before the report of the first case in-country/ region. See supplementary information for non-pharmaceutical interventions (NPIs) other than social distancing (SD).

SD measures have been implemented to different degrees by countries and regions affected by the COVID-19 pandemic. The beginning of this pandemic coincided with the Lunar New Year holiday and winter break in China, for which schools and workplaces were scheduled to close on 17 January and 24 January 2020, respectively. Due to the outbreak in Wuhan, stringent SD measures including intensive travel restrictions were introduced in the city on 23 January 2020. In response to the COVID-19 pandemic, school closures across China have been extended and remain in place as of 21 March 2020 ^13^. Outside of mainland China, Japan and South Korea reported the first cases of COVID-19 on 20 January 2020 ^9, 14^. This was followed quickly by cases reported in Hong Kong (23 January), Singapore (24 January), and Italy (31 January). In response to these first exported cases, case isolation and contact tracing were implemented by each region or country. Figure 1 shows the timing of interventions in different countries and regions relative to the reported cases over time. The date of the first reported case is also shown to represent the start of contact tracing and case isolation of exported cases.

At the time of analysis, the most commonly implemented SD measures in Wuhan (Hubei, China) and the five countries and regions reporting the highest COVID-19 case numbers outside of mainland China, were school closures followed by remote working and quarantine. Table 2 summarises the SD measures. We found a substantial variation in the timing and type of SD measures adopted by different countries and regions outside of mainland China. Notably, Singapore had implemented some partial SD measures even before the first in-country COVID-19 case was reported. We observed that countries affected most recently have implemented SD measures most rapidly and in quick succession. There were also differences in the degree to which SD measures, such as school closures, have been implemented. For example, within weeks, school closures in Japan which were initially implemented locally in a few affected schools were preemptively extended to the entire nation ^15^. We also observed that among non-SD measures, travel advisories and restrictions were the first NPIs implemented by each country or region (see Supplemental Table 1 for the most common non-SD measures and Supplemental Figure 1 for the timing of these interventions in different countries and regions relative to the reported cases over time; extended data ^10^).

**Table 2.** Summary of social distancing interventions implemented in Wuhan City, China and the 5 countries or regions reporting the highest number of COVID-19 cases. Countries and regions considered here are: Hong Kong, Italy, Japan, Singapore, South Korea and Wuhan. Many countries have been implementing quarantine measures of travellers.

Intervention type	Number of regions that have implemented	National (%)	Enforced (%)	Regions
Crowding	6	33.3%	100%	Hong Kong, Italy, Japan, Republic of Korea, Singapore, Wuhan
School closures	6	50%	66.7%	Hong Kong, Italy, Japan, Republic of Korea, Singapore, Wuhan
Quarantine	5	60%	80%	Hong Kong, Italy, Japan, Republic of Korea, Singapore
Workplace closures	5	60%	80%	Hong Kong, Italy, Japan, Republic of Korea, Wuhan
University closures	3	100%	100%	Italy, Republic of Korea, Wuhan
Contact tracing	2	100%	100%	Japan, Singapore
Isolation	2	100%	100%	Hong Kong, Singapore
Public communications	1	100%	0%	Hong Kong
Travel restrictions	1	0%	100%	Wuhan

## Discussion

SD measures have been implemented to different degrees by countries and regions affected by the COVID-19 pandemic. Interventions have been most stringent in Hubei province (China), where intensive travel restrictions have affected 40–60 million residents ^16, 17^. Across other parts of China, extensive public health efforts including quarantine, cancellation of large gatherings, and travel restrictions have been implemented ^18^. Outside of mainland China, countries and regions most affected by COVID-19 have or have started to introduce SD interventions in efforts to contain and limit the spread of COVID-19. For example, Singapore has conducted extensive contact tracing and quarantine measures for confirmed cases and Italy has enforced nationwide school closures ^19^.

The timing and the degree to which SD measures have been implemented varied between the countries and regions we considered, but also globally. For example, some countries and regions such as the USA have implemented reactive and selective local school closures only, whereas Hong Kong, for similar cumulative case counts, has introduced a large number of voluntary (e.g. advice on avoiding crowded places) and mandatory (e.g. quarantine, contact tracing, wide-scale proactive school closures) SD measures ^20^. It is important to note that most countries and regions have implemented isolation of cases, contact tracing and quarantine in response to the first imported cases from Hubei, China (Japan, Thailand, South Korea, USA, Singapore since mid- to late-January) ^9, 21^. Other countries have introduced interventions in response to a large number of newly reported cases (Italy and Iran) more recently ^22, 23^.

Many SD interventions have focused on public messaging to encourage positive behaviour change. For example, encouraging individuals to work remotely, avoid crowded areas, and restrict non-essential travel. As such interventions are not enforced, its effectiveness will be dependent on public compliance. A recent YouGov survey found that risk perception differed by country ^24^. A higher proportion of respondents in Asian countries reported being concerned about their risk of being infected compared to European or North American countries. This is also reflected in self-reported positive behaviour changes. A majority of respondents in Asia surveyed reported avoiding crowded places (e.g. 83% in Hong Kong). Advocating for remote working have led to the greatest positive behavior in mainland China and Hong Kong, with 67% and 45% reportedly avoiding going to the office, respectively. These high figures compared to other countries in Asia may be due to implementation of remote working for government offices.

Outside of Hubei province, China where the long-term implementation of substantial SD layered with the strict movement restrictions in Wuhan City and Hubei have reduced the reproduction number *R*_0_, estimated to be greater than 2 during the early stages of the outbreak, it is likely too early to be able to evaluate or quantify the true effectiveness of specific SD interventions on the epidemic in affected countries or regions ^25– 28^. Indeed as most countries have implemented a range of non-pharmaceutical measures such as travel restrictions, health screenings, and advice such as hand and cough hygiene intended to prompt behaviour change, it is difficult to quantify the effectiveness of SD in the absence of other control measures. However early studies have found that the relative effectiveness of case isolation and contact tracing was greater than travel restrictions or contact reduction ^18^. They additionally found that the rapid implementation of these combined NPIs, conducted one, two, or three weeks earlier could have reduced case numbers by 66%, 86%, and 95%, respectively up to three months from their introduction. However, the impact that these NPIs beyond May 2020 remains unknown.

Studies from pandemic influenza have also shown that the timing and duration of SD interventions will impact its effectiveness. For example, for influenza there are restricted benefits to time-limited interventions, with the potential reduction in mortality by up to 30% being eroded if the control was applied too late or lifted too early ^29^. When considering targeted layered containment strategies, Ferguson *et al.* found that the effectiveness of social distancing, rapid case ascertainment, and targeted prophylaxis were similar, with school closures playing an important role in each scenario, especially if values of R0 were ≤2 ^8^. A systematic review of the effectiveness of SD measures for pandemic influenza identified varying levels of evidence for avoiding crowding, workplace measures, and case isolation in the community ^18^. These particular SD measures are more resource intensive and are socially and economically disruptive. For COVID-19 most isolation has thus far been in a hospital setting. As more cases are reported in the community, protocols around case isolation may change towards voluntary home isolation or household quarantine. Household quarantine for influenza was found to have an overall effect, but within an affected household could increase risk of infection amongst quarantined individuals. Other resource intensive measures such as contact tracing were found to be effective in reducing influenza transmission when used in combination with other interventions such as quarantine and isolation. However this is not feasible in all settings or sustainable beyond the early phase of an epidemic when there are fewer cases. For influenza where children are known to be important for transmission as they are more susceptible to infection, are more infectious, and contribute to higher person-to-person contact rates, there was evidence that school closures could have a substantial effect on reducing transmission. However, the role of children in transmission of COVID-19 is still unknown. If children have the same or similar role in transmission as for influenza, then we could expect the same level of impact as has been estimated for influenza.

However, across all SD measures the most important consideration is the feasibility of its long-term implementation. The most effective measures in terms of stopping transmission, for example the lockdown of entire cities as implemented in Hubei province, are also the most socially and economically disruptive ^5^. As many measures start to be lifted across cities in China, as transmission has effectively been paused, we may observe a bounce-back effect. Countries and regions are therefore faced with the difficult task of balancing economically and socially sustainable and acceptable control measures which are likely to have the largest overall impact with the need to control growing case numbers.

## Data availability

### Underlying data

Zenodo: seabbs/CovidInterventionReview: Initial release. http://doi.org/10.5281/zenodo.3730771 ^12^

This project contains the following underlying data:

- output-data
  ∘ counts.csv (Daily case counts for the countries/regions considered)
  ∘ first-cases.csv (Date of first notified case by countries/regions considered)
  ∘ interventions.csv (A compiled list of categorised interventions in the countries/regions considered)
  ∘ summarised-non-social-distancing-ints.csv (Summary of non-social distancing measures)
  ∘ summarised-social-distancing-ints.csv (Summary of social distancing measures)

Data are available under the terms of the Creative Commons Zero "No rights reserved" data waiver (CC0 1.0 Public domain dedication).

### Extended data

Figshare: Adoption and impact of non-pharmaceutical interventions for COVID-19. https://doi.org/10.6084/m9.figshare.12037512.v1 ^10^

This project contains the following extended data:

- Adoption and impact of non-pharmaceutical interventions for COVID-19 Supplementary information.docx (Document containing supplementary figure and table)

Data are available under the terms of the Creative Commons Attribution 4.0 International license (CC-BY 4.0).

Replication code is available from GitHub

Replication code: https://github.com/seabbs/CovidInterventionReview

Archived replication code: http://doi.org/10.5281/zenodo.3730771 ^12^

License: Creative Commons Zero "No rights reserved" data waiver (CC0 1.0 Public domain dedication)
