# Supplementary material for: Adoption and impact of non-pharmaceutical interventions for COVID-19
Source: Wellcome Open Res. 2020 Apr 2;5:59. [Version 1] doi: 10.12688/wellcomeopenres.15808.1 (PMC7255913; doi:10.12688/wellcomeopenres.15808.1)
Supplement: Peer review history

# Peer review: Reviewer response for version 1

**DOI:** 10.21956/wellcomeopenres.17336.r38326

**Published:** 2020-06-01

**Authors:** Alyssa Bilinski

**Affiliations:** 1 Interfaculty Initiative in Health Policy, Harvard Graduate School of Arts and Sciences, Cambridge, MA, USA

This study provides a useful summary of non-pharmaceutical interventions implemented early in the COVID-19 pandemic. The authors collated data about different non-pharmaceutical interventions implemented in Wuhan, South Korea, Japan, Hong Kong, Singapore, and Italy and plotted these compared to case growth. During the rapidly changing pandemic, the authors’ curation of data and framing remain useful for thinking about the basket of available interventions and different times at which they may be applied. 

**Major comments**

1. While the paper is entitled “Adoption and impact of non-pharmaceutical interventions for COVID-19”, there does not appear to be much analysis of the impact of interventions. As authors note, it is difficult to develop a causal identification strategy when multiple interventions were being implemented simultaneously, but without further analysis, I would move away from the framing as an impact assessment.
2. I was slightly confused by the paper’s framing of the analysis as all non-pharmaceutical interventions vs. specifically social distancing measures. The authors separate travel measures from social distancing measures in the introduction’s 4 categories, but describe “stringent SD measures including intensive travel restrictions” in the results section. (I assume that the latter may refer to local ‘lockdown’ while the former is out-of-area travel, but this would be useful to clarify). Most of the methods and results sections seem to focus only on social distancing; other NPIs do not appear in figures or tables, and are only mentioned in one sentence of the results section. While these are clearly harder to quantify, it may be clearer to frame the paper as social distancing-focused?
3. I had some trouble following Figure 1, as there are many subtly different colors. It may be more informative to select a smaller number of interventions and/or annotate these with letter abbreviations. I also found the local/national distinction confusing to interpret on this plot, particularly when multiple lines overlapped.

**Minor comments**

1. While the authors note that selected countries were “among the first or most affected places outside of mainland China”, it might be useful to specify a more formal selection criteria, if this was applied. Likewise, were NPI types pre-specified before searching?
2. Unless I am misreading the colors, surprisingly few countries seem to incorporate case isolation.That seems odd and contrary to my understanding of epidemic control in e.g. Wuhan, unless I am misunderstanding the criteria for that intervention.

Is the work clearly and accurately presented and does it cite the current literature?

Yes

If applicable, is the statistical analysis and its interpretation appropriate?

Partly

Are all the source data underlying the results available to ensure full reproducibility?

Yes

Is the study design appropriate and is the work technically sound?

Partly

Are the conclusions drawn adequately supported by the results?

Yes

Are sufficient details of methods and analysis provided to allow replication by others?

Yes

Reviewer Expertise:

Health economics, infectious disease modeling.

I confirm that I have read this submission and believe that I have an appropriate level of expertise to confirm that it is of an acceptable scientific standard, however I have significant reservations, as outlined above.

# Peer review: Reviewer response for version 1

**DOI:** 10.21956/wellcomeopenres.17336.r38721

**Published:** 2020-05-26

**Authors:** Sheree M S Smith

**Affiliations:** 1 School of Nursing and Midwifery, Western Sydney University, Penrith, NSW, Australia

Thank you for the invitation to review this manuscript associated with non-pharmaceutical interventions (NPIs) utilised to limit the spread of SARS-CoV2 during this current pandemic. The obvious differences between this viral pandemic and recent influenza pandemics are that vaccines are not available and to date, pharmaceutical interventions such as anti-viral therapies have not been proven in the treatment of COVID-19. Irrespective of when a pharmaceutical therapy or vaccine becomes available, NPIs will continue to have a role in interrupting the spread of viral illnesses, locally, nationally and internationally. 

 The importance of *Imai et al's* paper centres on role of NPIs and specifically social distancing measures that have been implemented and the subsequent number of COVID-19 cases reported. Previous published research associated with influenza NPIs tends to separate social distancing measures from home quarantine and isolation of infected individuals. The separation of these two NPI health behaviours (social distancing and isolation) may centre on the issue that the former tends to reflect policy or action by government on activities where large numbers of people may gather such as schools, shopping malls and places of worship and the latter as a public health person-specific action ( CDC,2020; Aiello,2009 ^2^; Jefferson, 2011 ^1^).

 The strength of this paper is that it provides a timeline of the implementation of social distancing measures across a number of countries (Figure 1). Whilst travel restrictions are briefly mentioned, a detailed description of what these restrictions entailed may be of interest as the Wuhan travel restrictions were primarily between cities and across borders. Understanding changes to local public transport and whether social distancing measures associated with public transport and/or increased cleaning procedures at stations and on buses and trains were implemented, may be important in understanding community transmission. Culture may also play a role in adherence to social restrictions if the public health message is not tailored for that community or the threat is not perceived at an individual or societal level. Furthermore, understanding the community’s acceptance of previous public health messaging during other recent pandemics may also inform our understanding of social distancing, community behaviour and transmission during this current pandemic.

*Recommendations:*

- Definitions for social distancing differ between public health authorities in various countries and this manuscript may benefit from acknowledgement of these differences.
- In both table 1 and table 2 recommend removing contract tracing as a social distancing measure.
- There has been a number of published systematic reviews on NPIs that may inform the discussion. The incorporation of findings from well conducted reviews may enhance the interpretation of social distancing measures and their effectiveness during this current pandemic.

*References:*

 Jefferson T, Del Mar CB, Dooley L, Ferroni E, Al‐Ansary LA, Bawazeer GA, van Driel ML, Nair NS, Jones MA, Thorning S, Conly JM. Physical interventions to interrupt or reduce the spread of respiratory viruses. Cochrane Database of Systematic Reviews 2011, Issue 7. Art. No.: CD006207. DOI: 10.1002/14651858.CD006207.pub4. ^1^

 Aiello AE, Coulborn RM, Aragon TJ, et al. Research findings from nonpharmaceutical intervention studies for pandemic influenza and current gaps in the research. Am J Infect Control. 2010;38(4):251‐258. doi:10.1016/j.ajic.2009.12.007 ^2^

 Centers for Disease Control and Prevention (2020) Coronavirus Disease 2019 – Social distancing updated 6 May 2020

https://www.cdc.gov/coronavirus/2019-ncov/prevent-getting-sick/social-distancing.html

Is the work clearly and accurately presented and does it cite the current literature?

Yes

If applicable, is the statistical analysis and its interpretation appropriate?

Yes

Are all the source data underlying the results available to ensure full reproducibility?

Yes

Is the study design appropriate and is the work technically sound?

Yes

Are the conclusions drawn adequately supported by the results?

Yes

Are sufficient details of methods and analysis provided to allow replication by others?

Yes

Reviewer Expertise:

health services research, models of care for lung health, influenza NPIs

I confirm that I have read this submission and believe that I have an appropriate level of expertise to confirm that it is of an acceptable scientific standard.

## References

[1] : Physical interventions to interrupt or reduce the spread of respiratory viruses. *Cochrane Database Syst Rev*.2011; 10.1002/14651858.CD006207.pub4 CD006207 10.1002/14651858.CD006207.pub4 21735402PMC6993921

[2] : Research findings from nonpharmaceutical intervention studies for pandemic influenza and current gaps in the research. *Am J Infect Control*.2010;38(4) : 10.1016/j.ajic.2009.12.007 251-8 10.1016/j.ajic.2009.12.007 20226569

# Peer review: Reviewer response for version 1

**DOI:** 10.21956/wellcomeopenres.17336.r38722

**Published:** 2020-05-15

**Authors:** Núria Torner

**Affiliations:** 1 Department of Medicine, University of Barcelona, Barcelona, Spain

Thank you for inviting me to review this study of utmost importance right now. NPI have been implemented before in influenza pandemics, yet this new coronavirus virus offers some differences that could diminish NPI’s effectiveness. And, until an effective vaccine and treatment are available to treat those at risk of severe illness, these are the only tools at hand to slow down transmission and keep healthcare facilities within a reasonable and affordable work load.

 Nevertheless the economic and social impact of NPI that take into account school closure, shutdown of economic activity related to travel and commodities such as restaurants, non-essential retailers and so on, also has to be carefully measured to ensure a balance that is feasible to cope with.

 The critical importance of children and teenagers in transmission of influenza is well described, but is it so for SARS CoV-2? Instead nursing homes and elderly dwelling facilities have been shown to be the target for this virus, so in the face of a second wave or a new winter season comeback, it will be important to focus on these latter facilities to stop transmission and spare hospitalizations of severely ill patients that overload ICUs. So it might be more effective to implement strict NPIs instead of school closure that is highly disruptive for the community.

 Implementation of social distancing strategies is challenging indeed and if compliance with the strategy is high, hopefully transmission in a community can be averted. However, if neighboring communities do not also use these interventions, infected neighbors will continue to introduce the virus. In all, this research is very important to deliver at this time so it can be used as guideline to take the confinement decisions.

 As to specific considerations to this paper:

 Only a few countries/territories have been taken into account outside of mainland China, perhaps France and Spain could have also been added because the onset of exponential transmission did not differ that much from Italy and the NPI measures taken have been slightly different. Anyhow, according to Fig 1 the result of delay in implementation of NPI on a nationwide scale is quite evident.

 As to Fig 1, note that there is a different numerical expression in the vertical axis for Hong Kong, please delete the decimal point.

 The Wuhan city graph has local and national NPIs, but Wuhan is a city in the Hubei province. It’s not clear what is meant by the continuous and discontinuous line. So you mean that the entire territory of China adopted contact tracing and crowding NPIs bu mid January including Wuhan, and then only Wuhan and other regions locally implemented additional NPIs? There are two solid orange lines, is that correct, it’s a bit confusing, could you clarify this information by labeling accordingly?

 The paper focuses mainly on social distancing and closures, not on hygiene measures or use of masks, which has been implemented also and there have been many problems as to availability of effective protective equipment worldwide. The title should reflect this fact, instead it mentions NPIs as a whole. Also there is no formal impact assessment in the results section, so it is misleading to see the term in the tittle.

 Definitions for each social distancing measure (safety distance, stay-home, travel restrictions etc.) should be included in the Methods section. For example, the term Public communications is not clear as to what it refers to.

 Table 2 gives the enforcement % as a whole in all the territories/countries included in the study, this is okay for 100% enforcement, but for the lesser it would be nice to have it broken down to each country /territory.

 Only Wuhan implemented travel restrictions? Is this correct?

Is the work clearly and accurately presented and does it cite the current literature?

Yes

If applicable, is the statistical analysis and its interpretation appropriate?

Yes

Are all the source data underlying the results available to ensure full reproducibility?

Yes

Is the study design appropriate and is the work technically sound?

Partly

Are the conclusions drawn adequately supported by the results?

Partly

Are sufficient details of methods and analysis provided to allow replication by others?

Partly

Reviewer Expertise:

Epidemiology of infectious diseases and outbreak prevention and control.

I confirm that I have read this submission and believe that I have an appropriate level of expertise to confirm that it is of an acceptable scientific standard, however I have significant reservations, as outlined above.

# Peer review: Reviewer response for version 1

**DOI:** 10.21956/wellcomeopenres.17336.r38324

**Published:** 2020-04-27

**Authors:** Nancy Fullman

**Affiliations:** 1 University of Washington, Seattle, WA, USA

Thank you for the opportunity to review this interesting and timely study. In the absence of an effective vaccine or pharmaceutical measures against COVID-19, policies that fall within the non-pharmaceutical intervention (NPI) are vital for slowing viral spread and (hopefully) reducing burden.

 Each day brings updated and/or new knowledge to the COVID-19 evidence base, especially as more countries confront the virus and implement NPIs. Subsequently, any lapse of time between analysis, manuscript submission, and peer review inevitably involves a growing gap in what was initially included in any study. With this in mind, below are recommendations for improving the manuscript’s current content and analyses; the main decision point for the study’s authors is whether to expand this work to more accurately reflect the world’s current “Adoption and impact of non-pharmaceutical interventions for COVID-19” or to reframe the study’s content as a focus on a subset of locations within a certain time frame.

**Main recommendation 1: Update results to cover more countries and policies to date, *or* clearly frame this study as focusing on locations which had the earliest epidemics. **This submission provides an initial synthesis of NPIs adopted in a subset of locations (Wuhan City, Hong Kong, South Korea, Japan, Singapore, and Italy) initially affected by the novel coronavirus. It is not quite clear on when exactly this analysis occurred, and/or when the intake of new data was completed, but given the submission timing (April 2), and manuscript content (i.e., reference to cases as of 21 March 2020; Figure 1 having data through approximately March 10-15), it suggests that other locations – namely Spain, but also increasingly other countries in Europe (e.g., France, United Kingdom) and the United States – were also seeing rising COVID-19 cases and deaths at the time of analysis. Further, these locations had at least started implementing NPIs: in Spain, Madrid closed educational facilities on March 11 and non-essential services on March 13; many more communities in Spain saw similar closings from March 14-15. France had similar timelines at the national level, though initial mass gathering restrictions began on March 4. The US was much more fragmented at the state-level, but many types of NPIs were increasingly implemented after March 13 or March 16 (i.e., the time period at which national-level recommendations were more strongly endorsed by the US President). In sum, at the time of submission, it was likely that more locations (nationally and sub-nationally) had at least begun NPI implementation in response to COVID-19.

 The focus on locations with earliest COVID-19 epidemics is an understandable one; however, this focus was not clearly discernable until the Methods section, and it was definitely not conveyed in the Abstract (i.e., based on its reading and timing of submission, I had expected at least some information on US NPIs). Again – this is not an inherent flaw of the study’s foundation or analytic approach. Going forward, it should either be much clearer on its intent (i.e., a focus on *x* locations that reached *y* COVID-19 cases or deaths before *z* date), or expand the review and synthesis to reflect the current state of countries’ adoption and impact of NPIs for COVID-19.

**Main recommendation 2: More clearly define the types of NPIs included (and excluded) in this study.** As the authors rightly note, there are many types of NPIs that have been deployed against infectious diseases and epidemics more broadly, and particular types that have been used to date against COVID-19. However, the consistency and clarity with which the latter are described could be substantially improved. First, the study appears to be primarily focused on social distancing measures (also referred to as physical distancing measures) rather than a broader set of NPIs; this should be made more clear in the Title and Abstract so as to not set different expectations for readers. Otherwise, the broader set of NPIs should be consistently considered throughout the analysis. Second, what is included in Table 1 (summary of social distancing measures considered and/or implemented in response to COVID-19) do not fully correspond with those in Figure 1. Notably “public communications” is not clearly defined (Figure 1), which is likely because it is not considered a social distancing measure (and is primarily discussed in supplementary tables and figures); however, “public communications” is then included in Table 2’s “Summary of social distancing interventions implemented…”.

 Third, at least one key distancing measure appears to be omitted: stay-at-home orders, or the equivalent of ‘lockdowns’ and shelter-in-place as they were framed in many locations. Whether the policies expressly banned any kind of departure from an individual’s residence (or only during certain times and/or for certain types of essential activities), this type of social distancing policy was considered one of the most strict – and potentially impactful – for many of the locations included in this study. Isolation and quarantine are different than more blunt mandates to remain at home and not interact with others; isolation and quarantine are, in theory, supposed to be on the basis of confirmed or suspected infection (isolation), and then exposure to infected individuals (quarantine). Given the authors’ separation of the different types of educational facility closure, it also could be useful to consider mandates to close certain types of businesses (e.g., entertainment venues, recreational facilities) and then more broadly sweeping non-essential business closures. These policies were generally more focused on reducing contact and exposure of customers within business settings rather than the employees (which was the primary focus on workplace closures and advisories to work from home). Restaurant and bar restrictions, whereby such businesses had to ease in-person services but could provide food and/or drink for off-site consumption if proper public health measures were in place, are another type of social distancing policy that is not expressly included in the current manuscript. Last, it would be helpful to hear more about why the authors did not include travel restrictions as a type of social distancing policy. Depending on what the restriction aims to achieve, it could squarely fall within a social distancing measure definition (i.e., a policy or measure that aims to reduce mixing and rates of close human contact with each other). Based on the three-point definition of social distancing measure on page 3, it seems as if travel restrictions (e.g., banning on non-essential travel outside of your state or community of residence; requiring quarantine for all travelers arriving in location *x*, irrespective of points of origin) would fall under “community containment”.

**Main recommendation 3: Remove impact from the study’s title *or *conduct a more formalized synthesis of NPI/social distancing policy impact.** Because the manuscript’s title indicated that this was a study of “adoption and impact” of NPIs for COVID-19, there was an expectation that a more formal quantification of impact would be included. Of course such an analysis is fraught with challenges, especially when more stringent distancing policies may be implemented in response to worsening epidemic trajectories in many places (i.e., issues of endogeneity), and widespread gaps or delays in testing (i.e., issues with properly quantifying COVID-19 outcome measures). Increasingly more pre-print studies are using changes in human movement based to approximate the impact of social distancing measures and how changes in mobility might be related to subsequent changes in transmission and infection. Further, other studies are increasingly considering which types of social distancing policies have been related to larger changes in mobility (and thus potentially transmission) – a crucial step to understanding what combinations of measures may be most effective for a given setting and epidemiologic context. To most accurately retain “impact” in the study’s title would require at least some kind qualitative, and preferably quantitative, synthesis of the different types of studies conducted to date on the impact of social distancing measures in response to COVID-19.

**Other minor recommendations and queries:**

- Introduction: broader types of NPIs are discussed here (e.g., personal protective measures), but are not really revisited in the Discussion. Strengthening the ties between the different types of NPIs and when they might be best used throughout an epidemic’s course could be beneficial for this study.
- Results: Table 2: What does “Enforced” mean? By law, or by physical force (e.g., police, military)? Means of enforcement could certainly vary across the included locations.
- Discussion (page 4, right-hand column, second paragraph): even by mid-late March, the US had markedly expanded its social distancing policies beyond local school closures. Statewide orders started accelerating in mid-March, and various types of business closures increasingly occurred. California had the country’s stay-at-home order and non-essential business closure issued on March 19, followed by New York State. Discussing COVID-19 responses in other European countries (Spain, France, UK, among others) would be helpful in this section and elsewhere in the Discussion.
- Final paragraph (page 8): consider including information on the role of social distancing measures alongside the scale up of containment strategies (i.e., widely available testing, case-based isolation and contact tracing), especially in locations looking to ease distancing policies. Further, given the resurgence of cases in many of the places covered in this study post-easing of social distancing policies, what the implications of longer term use of NPIs?

Is the work clearly and accurately presented and does it cite the current literature?

Partly

If applicable, is the statistical analysis and its interpretation appropriate?

Partly

Are all the source data underlying the results available to ensure full reproducibility?

Yes

Is the study design appropriate and is the work technically sound?

Partly

Are the conclusions drawn adequately supported by the results?

Yes

Are sufficient details of methods and analysis provided to allow replication by others?

Yes

Reviewer Expertise:

Population health measurement, health systems, infectious diseases.

I confirm that I have read this submission and believe that I have an appropriate level of expertise to confirm that it is of an acceptable scientific standard, however I have significant reservations, as outlined above.
